# *Mycobacterium senegalense* Infection after Implant-Based Breast Reconstruction, Spain

**DOI:** 10.3201/eid2603.190230

**Published:** 2020-03

**Authors:** Octavio Carretero, Carmen Reyes, Rafael San-Juan, Fernando Chaves, Paula López-Roa

**Affiliations:** Hospital Universitario 12 de Octubre, Madrid, Spain

**Keywords:** Mycobacterium senegalense, mastectomy, prosthesis, breast implant, tuberculosis and other mycobacteria, bacteria, Spain

## Abstract

Bacterial infection is a well-known complication of breast implant surgery. We identified *Mycobacterium senegalense*, the principal pathogen of bovine farcy of cattle, in a woman after implant-based breast reconstruction. This finding indicates that unusual pathogens should be considered as an etiology of infected breast prostheses.

*Mycobacterium senegalense* is a rapidly growing mycobacterium that belongs to the *M. fortuitum* complex. These opportunistic pathogens cause posttraumatic skin and soft tissue infections, including postsurgical wound infections (*1*). *M. senegalense* is the principal pathogen of bovine farcy, which is endemic to eastern and central Africa (*2*). Few human cases have been described (*3*–*5*).

In 2014, a 44-year-old woman in Spain had a right breast mastectomy with lymph node dissection, radiotherapy and chemotherapy for breast carcinoma, followed by bilateral breast augmentation in November 2016. Her postoperative course was uneventful until January 2017, when a serous discharge appeared on the right breast. Consequently, she underwent removal of her right breast implant, drainage, and sample collection. Standard bacteriologic cultures were sterile.

In August 2017, the patient underwent repeat implant-based breast reconstruction. Three months after surgery, she sought care for edema and erythema of her right breast. Nuclear magnetic resonance imaging showed inflamed fistulous tracts. Serous fluid obtained during the implant surgery was sent to the microbiological laboratory at Hospital Universitario 12 de Octubre (Madrid. Spain). After 48 hours of incubation, the blood agar grew slow-growing tiny colonies identified as *M. senegalense* by using matrix-assisted laser desorption/ionization time-of-flight (MALDI-TOF) mass spectrometry (Bruker Daltonics, https://www.bruker.com) with a score of 2.1 (scores >1.8 are accepted for mycobacterial species assignment). Before MALDI-TOF mass spectrometry analysis, bacteria were heat-inactivated in a dry water bath at 95°C for 30 min and submerged in ethanol to a final concentration of 75%. The cells were then physically disrupted by using a protein-extraction protocol recommended by the manufacturer (MycoEx version 3.0; Bruker Daltonics).

We performed partial sequencing of the 16S ribosomal RNA and *rpoB* genes of the isolate (*6*). The partial sequence of 16S rRNA showed 100% identity to the sequences deposited in GenBank of 7 *Mycobacterium* species: *M. farcinogenes*, *M. senegalense*, *M. houstonense*, *M. conceptionense*, *M. fortuitum*, *M. mucogenicum*, and *M. phocaicum*. Partial *rpoB* gene sequencing showed 99% identity with the *M. senegalense* and *M. conceptionense* sequences. The latest update of MALDI-TOF mass spectrometry database includes *M. conceptionense*, but we did not find this species among the top matches. Therefore, using a combination of 16S rRNA/rpoB gene sequencing and MALDI-TOF mass spectrometry, we identified the isolate as *M. senegalense*. By using the Etest method, we determined the isolate was susceptible to cefoxitin, amikacin, ciprofloxacin, clarithromycin, trimethoprim/sulfamethoxazole, and minocycline.

After prostheses removal and debridement of the fistulous tract, an extended treatment was scheduled with moxifloxacin (400 mg/d for 4 weeks). The patient’s clinical course was uneventful until May 2018, when she sought care for fistulization of the surgical wound. *M. senegalense* exhibiting an identical antimicrobial drug susceptibility pattern was again isolated from the wound fluid. Nuclear magnetic resonance imaging showed bone impairment and new fistulous tracts. The patient was then treated over a 3-month period with a combination of clarithromycin (500 mg 2×/d) and trimethoprim/ sulfamethoxazole (160.800 mg 2×/d). After finishing the combination therapy, she completed 3 months of clarithromycin monotherapy. At a follow-up examination 6 months later, she had no signs or symptoms of infection, and ultrasound detected nothing relevant.

No clear habitat for human strains of *M. senegalense* outside of Africa has been reported. We do not know how this patient’s breast prosthesis became infected with *M senegalense*, but we speculate the infection occurred during implant-based breast reconstruction.

Limited studies have reported *M. senegalense* as the causal agent of human diseases (*3*–*5*). In the case reported here, the absence of other pathogens and its repeated isolation from clinical specimens suggests the clinical significance of this species.

MALDI-TOF mass spectrometry identified *M. senegalense* with a score of 2.1, which is considered high-confidence identification at species level. 16S rRNA sequencing did not enable us to discriminate between 7 closely related species. Although *rpoB* gene sequencing has higher discrimination power, it could not distinguish between *M. senegalense and M. conceptionense*. These findings suggest that the combination of 16S rRNA/rpoB partial gene sequencing and MALDI-TOF mass spectrometry is useful and reliable for identifying this species.

Because previous experience with this organism is limited, no specific treatment guidelines exist. Therefore, the treatment course was based on current recommendations for breast implant infection caused by *M. fortuitum*. Although some reports have suggested success with single-agent treatment, combination therapy is recommended to avoid the emergence of resistance. No standard duration of therapy is reported, and treatment may last >6 months (*6*–*8*). In this case, the complete removal of prosthetic material was considered curative, and an extended antimicrobial treatment with moxifloxacin was prescribed. After relapse, a combined treatment was administered, followed by prolonged treatment with clarithromycin, which was finally curative.

In conclusion, the case reported here is a reminder that unusual pathogens, such as *M. senegalense*, should be considered as an etiology of infected breast prosthesis. Molecular techniques confirmed the accuracy of MALDI-TOF mass spectrometry to identify this emerging mycobacterial species. Patients should undergo prolonged treatment for >3 months, ideally with combined therapy, even with adequate surgical treatment.
